# In This Issue: Volume 117, Issue 4, April 2026

**DOI:** 10.1111/cas.70370

**Published:** 2026-04-02

## Clinical Applications of Phosphoproteomics: Illuminating Cancer Signaling and Enabling Rational Therapeutic Strategies

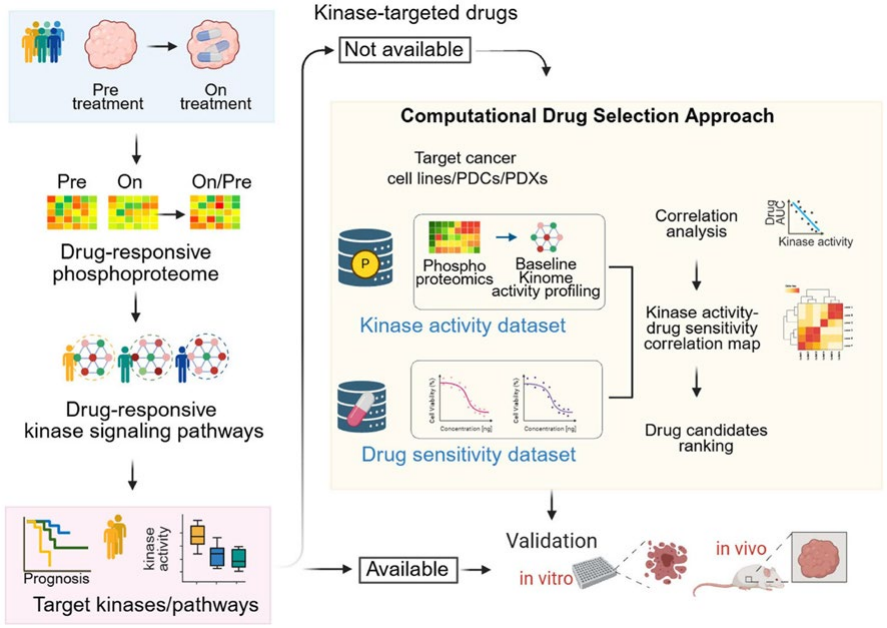

Cells rely on complex communication systems to control how they grow, divide, and survive. One important way this communication occurs is through a process called protein phosphorylation, in which small chemical tags are added to proteins. These tags act like switches that turn cellular signals on or off. When these signaling switches stop working properly, cells may begin to grow uncontrollably, which can lead to cancer. Understanding how these signaling changes occur inside cancer cells is therefore important for developing more effective treatments.

In this review, Shoji et al. describe how a research approach known as phosphoproteomics is helping scientists better understand cancer signaling. This method allows researchers to examine thousands of proteins at once and identify where phosphorylation occurs within them. Using advanced technologies such as mass spectrometry, scientists can now detect tens of thousands of phosphorylation sites in a single experiment. These technological advances also make it possible to analyze very small clinical samples, including tumor biopsies collected from patients.

By studying these phosphorylation patterns, researchers can build detailed maps of the signaling networks that operate inside cancer cells. These maps reveal how cancer cells alter normal communication systems to support their growth and survival. One key finding from such studies is the identification of enzymes called kinases, which control many phosphorylation signals. In several cancers, certain kinases become overly active and drive tumor progression. Identifying these enzymes can therefore help scientists discover promising targets for new cancer therapies.

Another advantage of phosphoproteomics is that it can reveal signaling changes that may not be detected through genetic studies alone. Together, the work highlighted by the authors suggests that phosphoproteomics could guide more precise and personalized cancer treatments.

https://onlinelibrary.wiley.com/doi/full/10.1111/cas.70323

## Therapeutic Strategies to Overcome Payload Resistance of Trastuzumab Deruxtecan in HER2‐Positive Cancers

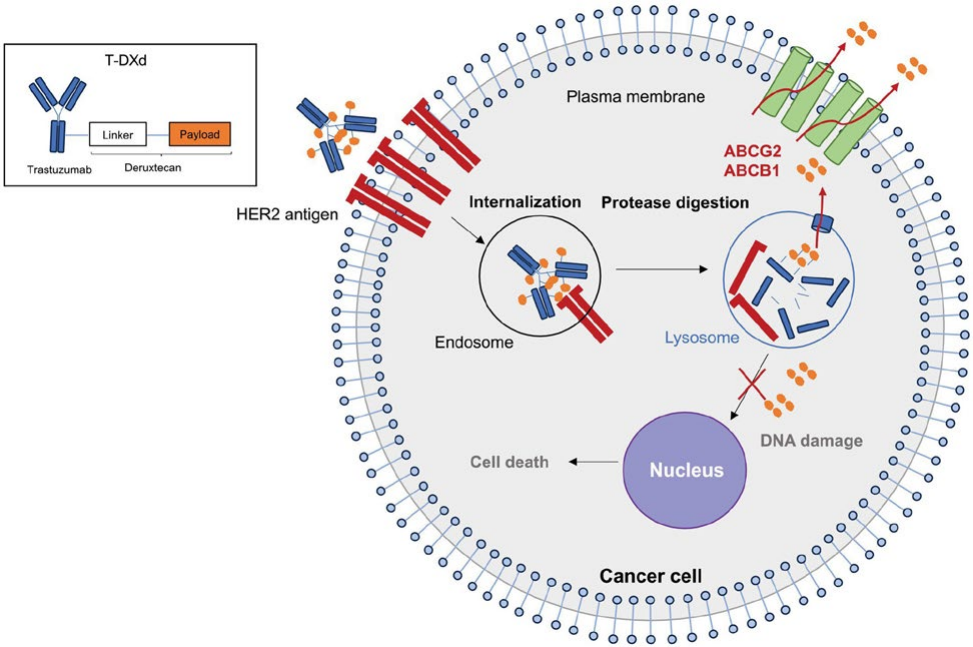

Antibody–drug conjugates (ADCs) are an emerging class of anticancer agents designed to attack tumors while limiting damage to healthy cells. Trastuzumab deruxtecan (T‐DXd), a human epidermal growth factor receptor 2 (HER2)‐directed ADC, has gained significant attention due to its promising results against certain breast, gastric, and lung cancers. However, acquired resistance in cancer cells causes many patients to eventually stop responding to this therapy, limiting the drug's long‐term efficacy.

To address this, Murase et al. conducted a study to identify the underlying mechanism through which these cancer cells acquire resistance to T‐DXd. The researchers created laboratory models of gastric and lung cancers that had become resistant to trastuzumab deruxtecan. They discovered that the resistant cancer cells still carried HER2 and continued to take up the drug normally. Instead, the cells had increased activity of specific transport proteins that pump harmful substances out of the cell. These proteins, known as ATP‐binding cassette (ABC) transporters, effectively removed the drug's toxic payload before it could damage the cancer cells. When the researchers blocked these transporters using genetic or chemical approaches, the cancer cells became sensitive to the treatment again. The team also tested another HER2‐targeted therapy carrying a different drug payload, which successfully killed the resistant cells.

Together, these findings suggest that drug‐pumping transporters play a key role in resistance to trastuzumab deruxtecan. Blocking these transporters or using alternative targeted drugs may help restore treatment effectiveness and provide new options for patients with HER2‐positive cancers.

https://onlinelibrary.wiley.com/doi/full/10.1111/cas.70319

## Roles of TIF1β in Leukemic Stem Cell Through SETDB1‐Dependent and Independent Mechanisms

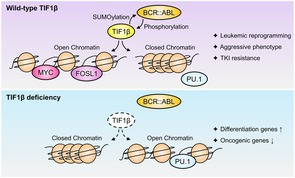

Normal growth and development of cells depend on the carefully controlled activation and silencing of genes. One mechanism for silencing genes is to fold specific sections of chromosomes into tightly packed regions called heterochromatin. Genes in heterochromatin cannot be read and expressed into proteins.

Transcriptional Intermediary Factor 1β (TIF1β) is a protein that induces the formation of heterochromatin. It is ubiquitously expressed across tissues and plays a critical role in embryonic development.

However, growing evidence suggests that TIF1β may interact with diverse partners and also contribute to cancer biology.

A recent review article by Morii et al. aimed to discuss TIF1β's physiological role in stem cell maintenance, differentiation, and immune regulation while also shedding light on its oncogenic role.

The authors noted that TIF1β undergoes five kinds of post‐translational modifications that facilitate its role as an epigenetic regulator. These functions include, but are not limited to, antibody production in bone marrow, muscle fiber growth in muscles, and release of defensive proteins by the liver in response to an infection.

TIF1β usually works together with heterochromatin protein 1 (HP1) and the H3K9 methyltransferase SETDB1 to form a repressive complex that silences genes—by a process called histone methylation—necessary for preserving stem cell identity and stability.

However, the authors point out that under tumor‐promoting stress, TIF1β transitions to a transcriptional co‐activator role, promoting gene expression and leukemia progression. High TIF1β is linked to the expression of genes that sustain undifferentiated stem‐like states, prevent programmed cell death, and protect from cell death caused by the immune system's T cells. As a result, high TIF1β in tumors often indicates that the cancer will grow aggressively and will likely be resistant to treatment. Similarly, suppressing TIF1β in cancerous cells slows their growth.

It appears that TIF1β's function depends on other proteins in its environment. In healthy cells, TIF1β silences genes to maintain normal functions. However, in the presence of mutant, cancer‐causing proteins, TIF1β switches to activating other cancer‐sustaining genes, demonstrating its epigenetic plasticity. The authors conclude that silencing TIF1β selectively in cancerous tissue, especially in the bone marrow, could be a promising new avenue for therapies against leukemia.

https://onlinelibrary.wiley.com/doi/full/10.1111/cas.70334

